# Influence of YES1 Kinase and Tyrosine Phosphorylation on the Activity of OCT1

**DOI:** 10.3389/fphar.2021.644342

**Published:** 2021-03-08

**Authors:** Muhammad Erfan Uddin, Dominique A. Garrison, Kyeongmin Kim, Yan Jin, Eric D. Eisenmann, Kevin M. Huang, Alice A. Gibson, Zeping Hu, Alex Sparreboom, Shuiying Hu

**Affiliations:** ^1^ Division of Pharmaceutics and Pharmacology, College of Pharmacy and Comprehensive Cancer Center, The Ohio State University, Columbus, OH, United States; ^2^ School of Pharmaceutical Sciences, Tsinghua University, Beijing, China

**Keywords:** organic cation transporter 1, YES1 kinase, tyrosine kinase inhibitors, drug-transporter interactions, post-translational modification

## Abstract

Organic cation transporter 1 (OCT1) is a transporter that regulates the hepatic uptake and subsequent elimination of diverse cationic compounds. Although OCT1 has been involved in drug-drug interactions and causes pharmacokinetic variability of many prescription drugs, details of the molecular mechanisms that regulate the activity of OCT1 remain incompletely understood. Based on an unbiased phospho-proteomics screen, we identified OCT1 as a tyrosine-phosphorylated transporter, and functional validation studies using genetic and pharmacological approaches revealed that OCT1 is highly sensitive to small molecules that target the protein kinase YES1, such as dasatinib. In addition, we found that dasatinib can inhibit hepatic OCT1 function in mice as evidenced from its ability to modulate levels of isobutyryl L-carnitine, a hepatic OCT1 biomarker identified from a targeted metabolomics analysis. These findings provide novel insight into the post-translational regulation of OCT1 and suggest that caution is warranted with polypharmacy regimes involving the combined use of OCT1 substrates and kinase inhibitors that target YES1.

## Introduction

In the last two decades, considerable advances have been made toward understanding the pharmacological role of cationic transporters belonging to the SLC22A subfamily. The advent of heterologous overexpression systems and genetically-engineered murine models has substantiated that the members of this subfamily facilitate the cellular uptake of a large number of structurally diverse endogenous metabolites and an increasingly large number of cationic xenobiotics. Organic cation transporter 1 (OCT1, *SLC22A1*) is the most abundant cationic transporter expressed on the sinusoidal membrane of hepatocytes (Chen et al., 2014), and is a rate-limiting step in the sodium-independent uptake and elimination of many xenobiotic substrates (Koepsell et al., 2007; Shu et al., 2007; Huang et al., 2020).

The *in vivo* contribution of OCT1 to the hepatic elimination of xenobiotics was first conclusively demonstrated for the prototypical organic cation, tetraethylammonium (TEA), in mice harboring a genetic deletion of OCT1 (Jonker et al., 2001; Jonker et al., 2003). Many subsequent studies have focused on the biguanide analog metformin, a first-line medication for the treatment of type 2 diabetes. These studies have led to the recognition that the glucose-lowering effects of metformin are partially dependent on OCT1 (Chae et al., 2016; Heckman-Stoddard et al., 2016), and that OCT1 deficiency is associated with diminished metformin uptake in hepatocytes (Shu et al., 2007; Higgins et al., 2012). More recently, OCT1 has also been identified as a critical determinant of the therapeutic efficacy of fenoterol (Tzvetkov et al., 2018), morphine (Fukuda et al., 2013), sumatriptan (Matthaei et al., 2016), thiamine (Chen et al., 2014), tramadol (Tzvetkov et al., 2011), and tropisetron (Tzvetkov et al., 2012).

Due to its predominant role in determining the efficacy of many clinically-important drugs, multiple regulatory aspects of OCT1 have been widely studied. For example, polymorphic variants in OCT1 (Gomez and Ingelman-Sundberg, 2009) have been linked to the pharmacokinetics and glycemic response in diabetic patients receiving metformin (Giacomini et al., 2012), and epigenetic mechanisms have been identified that can functionally modulate OCT1 and can profoundly affect therapeutic outcomes of substrate drugs (Schaeffeler et al., 2011). Although post-translational modification *via* phosphorylation has been reported to influence the function of transporters (Mehrens et al., 2000; Czuba et al., 2018), surprisingly, this has not been extensively studied as a regulatory mechanism of OCT1. We previously reported that the related transporter OCT2 (*SLC22A2*) is sensitive to inhibition by several FDA-approved tyrosine kinase inhibitors (TKIs) through a mechanism that involves YES1-mediated tyrosine phosphorylation (Sprowl et al., 2016). Since OCT1 and OCT2 share structural features, a high degree of sequence homology, and have overlapping substrate recognition sites and conserved tyrosine motifs (Tanaka and Herr, 1990; Gorboulev et al., 1997), we hypothesized that the activity of OCT1 is also dependent on kinase-mediated tyrosine phosphorylation. In the current study, we tested this hypothesis by employing phospho-proteomics screens, genetic strategies, pharmacological approaches, and metabolomics analyses in heterologous models overexpressing mouse or human OCT1, as well as OCT1-deficient mice.

## Materials and Methods

### Cell Culture and Reagents

Human embryonic kidney (HEK293) cells were obtained from the American Type Culture Collection (ATCC, Manassas, VA). HEK293 cells stably transfected with mouse OCT1 (mOCT1) or human OCT1 (hOCT1) were cultured in Dulbecco’s Modified Eagle Media (DMEM) media supplemented with 10% fetal bovine serum (FBS) and grown at 37°C in a humidified incubator containing 5% CO_2_. Radiolabeled [^14^C] TEA and [^14^C] metformin were obtained from American Radiochemicals (St. Louis, MO). Cellular uptake assays were performed 48 h following transient transfection by Lipofectamine 3000 Transfection Reagent (Thermo Fisher Scientific, Waltham, MA). ON-TARGETplus Human YES1 siRNA was obtained from Dharmacon (Lafayette, CO). RNA extraction kits were obtained from Omega Bio-tek (Norcross, GA). Reference standards of decynium22, a positive control inhibitor, as well as the TKIs bosutinib, dasatinib, gilteritinib, ibrutinib, lapatinib, sunitinib, vandetanib, and CH6953755 were obtained from MedChemExpress (Monmouth Junction, NJ).

### Cellular Accumulation Studies

Uptake assays were performed with radiolabeled TEA (2 µM) or metformin (5 µM) as described previously (Sprowl et al., 2013; Pabla et al., 2015) in the presence or absence of TKIs. The results were normalized to uptake values in cells stably transfected with an empty vector treated with vehicle alone (Supplementary Figure S1). Prior to cellular accumulation experiments, cells were grown to 90% confluence on poly-lysine coated multi-well plates. For uptake studies, cells were rinsed with warm PBS and incubated in the presence of a vehicle or inhibitor, prepared in serum and phenol red-free DMEM media for 15 min. Subsequently, media was removed followed by the addition of radiolabeled TEA and metformin along with inhibitor, and cellular uptake was measured after a 15-min co-incubation period. Total radioactivity originating from TEA and metformin was determined using liquid scintillation counting after lysing the cells with 1 N NaOH, a neutralizing step with 2 M HCl. A Pierce protein assay (Thermo Fisher Scientific, Waltham, MA) was used to normalize radioactivity readings to account for variation in cell number between samples.

### Site-Directed Mutagenesis

The YES1 plasmid with pCMV6-Entry (C-terminal FLAG-tagged) backbone was obtained from Origene (Rockville, MD). Mutants in OCT1 and YES1 were generated using QuikChange XL Site-Directed Mutagenesis Kit (Agilent Technologies, Santa Clara, CA). Mutagenesis primers were designed using QuikChange Primer Design software and generated according to the manufacturer’s instructions. Successful mutagenesis was confirmed by Sanger sequencing and constructs used for transient transfection experiments.

### siRNA-Mediated Knockdown

HEK293 cells overexpressing hOCT1 were plated at a density of 1.25 × 10^5^ per well in a 12-wells plate and incubated overnight at 37°C with 5% CO_2_. The next day, cells were transfected with 50 nM siRNA targeting YES1, positive control siRNA, and negative control siRNA (Dharmacon, Lafayette, CO) according to manufacturer protocols. After 48 h of exposure to siRNA, OCT1 function was evaluated with TEA or metformin as described above.

### RT-qPCR

Total RNA was extracted from cells treated with siRNA by E.Z.N.A. Total RNA Kit I (Omega Bio-tek), and reverse transcribed to cDNA by qScript XLT cDNA SuperMix (QuantaBio, Beverly, MA). Primer sequences included YES1 (Hs00736972_m1) and human GAPDH (Hs02758991_g1), and quantitative RT-PCR was performed using TaqManTM Fast Advanced Master Mix (Thermo Fisher Scientific, Waltham, MA). The Ct values of the YES1 gene were subtracted from the mean of GAPDH (ΔCt). All samples were analyzed in triplicate, and the mean value of ΔCt was calculated.

### Protein Analysis

Cell treated with non-targeting siRNA and YES1 siRNA were lyzed using sonication. Pierce Bicinchoninic Acid (BCA) Protein Assay Kit (Thermo Fisher Scientific, Waltham, MA) was used to determine protein concentrations. Next, an equal amount of protein was separated on a Bis-Tris 4–12% SDS-polyacrylamide gel with MOPS buffer according to the instructions from manufacturer (Life Technologies, Grand Island, NY) and transferred to PVDF membranes. Western blot analysis was performed using antibodies against YES1 (Product # 3201S), vinculin (Product # 13901S), and HRP-conjugated secondary anti-rabbit (Product # 7074) obtained from Cell Signaling Technology (Danvers, MA). Proteins were visualized by chemiluminescence using the SignalFire ECL Reagent (Cell Signaling Technology, Danvers, MA) or SuperSignal West Femto Maximum Sensitivity Substrate (Invitrogen, Carlsbad, CA) using film.

### Proteomics and Metabolomics Studies

In order to evaluate the tyrosine-phosphorylation landscape of ADME proteins in FVB mice, the genetic background strain used in our transporter-deficient *in vivo* models, tissue samples were subjected to a PhosphoScan analysis (Cell Signaling, Danvers, MA). This analysis provides purification and characterization of tyrosine phosphorylation sites in cellular proteins when paired with liquid chromatography tandem mass-spectrometry (LC-MS/MS) technology. The assay comprises enhanced phospho-tyrosine-containing peptides using P-Tyr-100, a mouse anti-phospho-tyrosine antibody paired with protein G agarose beads. Following protease-mediated digestion, immune-affinity purify-cation of peptides, and MS analysis on phospho-peptides, spectra were assessed using Sequest 3G and the Sorcerer 2 platform (Sage-N Research, Milpitas, CA).

For metabolomics studies, plasma and tissue samples were collected from wild-type mice and OCT1/OCT2 (OCT1/2)-deficient mice (Taconic, Petersburgh, NY). Tissue samples were washed with ice-cold 0.9% saline, and snap-frozen using liquid nitrogen. Further preparation of plasma and tissue samples for metabolomics analysis was done using LC-MS/MS, as previously described (Huang et al., 2018).

### Animal Experiments

For all *in vivo* studies, plasma and tissue samples were collected from both males and females wild-type mice, OCT1/2-deficient mice, and mice additionally deficient for MATE1 (OCT1/2/MATE1), following an established protocol (Leblanc et al., 2018). Mice were maintained under pathogen-free conditions at the Ohio State University Laboratory Animal Resources, and all *in vivo* experiments were approved by University Animal Care and Use Committee (protocol number: 2015A00000101-R1). Mice were accommodated in a temperature-, and light-controlled environment with access to water and food. OCT1/2/MATE1-deficient mice was obtained by crossing male OCT1/2-knockout mice with female MATE1-knockout mice to generate heterozygous breeders. The MATE1-deficient mice used to generate this model were kindly provided by Dr. Yan Shu (University of Maryland, Baltimore, MD), and backcrossed onto an FVB background. Next, heterozygous males and females were used to obtain OCT1/2/MATE1-knockout mice. Genetic deletion of OCT1/2 and MATE1 was confirmed by performing RT-PCR analysis.

Dasatinib was dissolved in 80 mM citric acid (pH 3.1) and administered *via* oral gavage at a dose of 15 mg/kg. For studies involving TEA, dasatinib was given orally 30 min before the intravenous administration of [^14^C] TEA (0.2 mg/kg) *via* the caudal vein. Concentrations of total TEA-derived radioactivity in plasma and homogenized liver samples were measured by liquid scintillation counting.

### Quantification of Isobutyryl L-Carnitine (IBC)

A Vanquish UHPLC paired with a Quantiva triple quadrupole mass spectrometer (Thermo Fisher Scientific) was used to perform LC-MS/MS analysis of IBC and the internal standard, isobutyryl L-carnitine-d3 (Cayman Chemical, Ann Arbor, MI). Chromatographic separation of analytes was achieved on an Accucore aQ column (150 mm × 2.1 mm, dp = 2.6 μm) with a C18 AQUASIL guard cartridge (2.1 mm × 10 mm, dp = 3 μm). The temperature of the column and autosampler was retained at 40 and 4°C, respectively. The mobile phase contains solvent A (0.1% formic acid in water) and solvent B (0.1% formic acid in acetonitrile-methanol, 50:50 v:v). The gradient elution was 5.0 min at a flow rate of 0.4 ml/min, and conditions were as follows: 0–0.5 min, 0% B; 0.5–2.3 min, 30% B; 2.3–3.8 min, 30–95% B; 3.8–4.2 min, 95% B; 4.2–5.0 min, 0% B. The extracted samples (5 μl) were injected for analysis, and following parameters were established for the mass spectrometer: 40 Arb, 12 Arb, 3.3 Arb, 350, and 375°C for sheath gas, aux gas, sweep gas, ion transfer tube, and vaporizer temperature, respectively. The ion source was managed by heated ESI in positive ion mode with ion spray voltage at 3,500 V. Argon was used as a collision gas at a pressure of 1.5 mTorr. Precursor molecular ions and product ions were recorded for confirmation and detection of IBC (232.144 > 85.083) and the internal standard (236.056 > 85.056). Assay validation studies demonstrated that the within-day precision and between-day precision ranged from 0 to 6.16%, and the accuracy ranged from 92.8 to 105%. The lower limit of quantification for IBC was 0.1 ng/ml.

### Statistical Analysis

All data are presented as mean ± SEM, either as the experimental readings or after normalization to baseline values, and then expressed as a percentage. All experiments were conducted in triplicate unless specified, and were performed on at least two independent occasions. Comparisons between two groups were analyzed by unpaired two-sided Student’s *t*-test with Welch’s correction while one-way ANOVA with Dunnett’s post-hoc test was performed for comparing more than two groups. Statistical analyses were conducted using GraphPad Prism version 8.1.2 (GraphPad Software, San Diego, CA), and *p* < 0.05 was considered as the cutoff for statistical significance.

## Results

### Conserved Tyrosine Phosphorylation of OCT1

**FIGURE 1:**
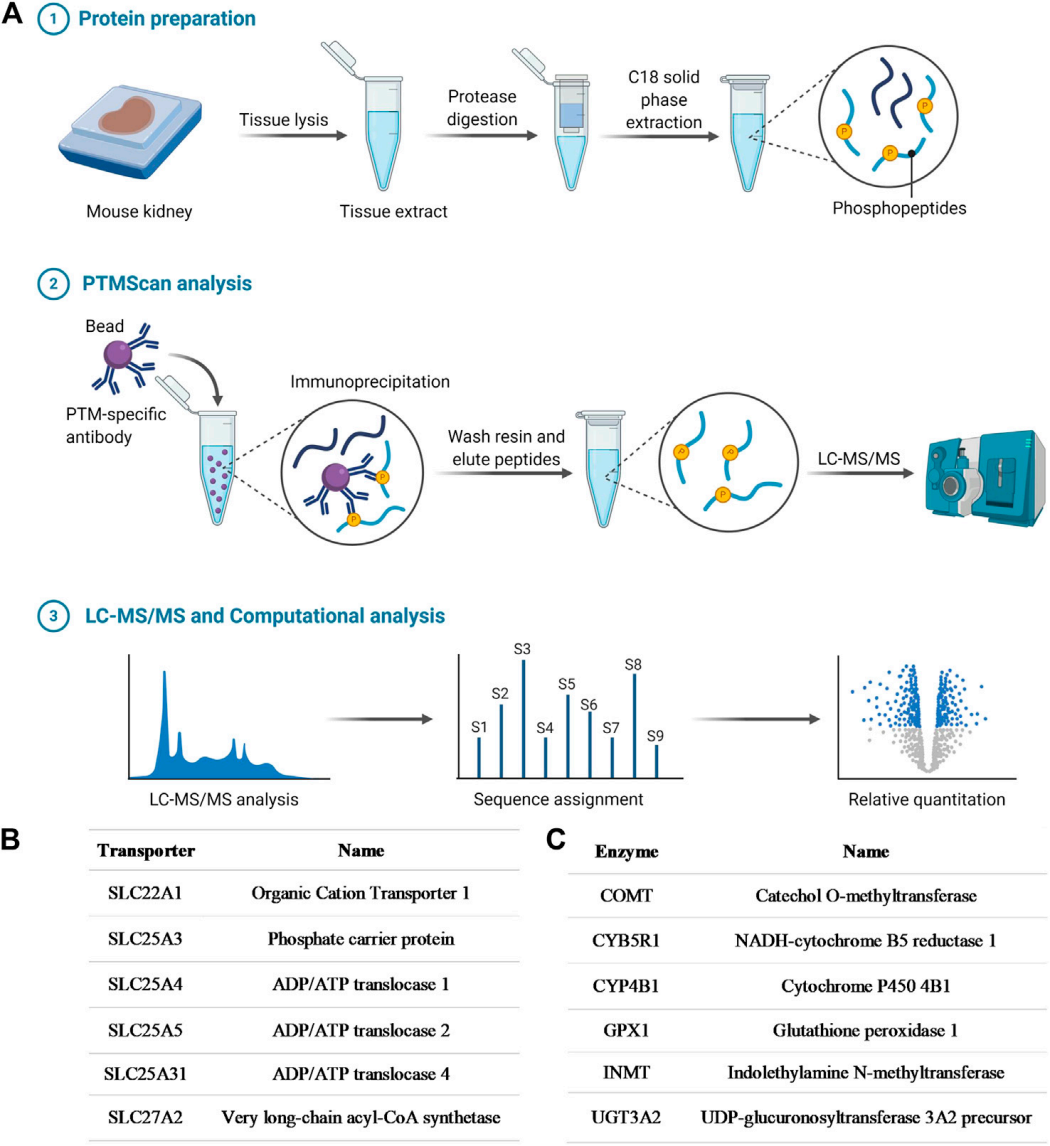
Phosphotyrosine proteomics screen. **(A)** Schematic diagram depicting the PhosphoScan analysis from wild-type mouse kidney samples. Identified SLC transporters **(B)** and enzymes **(C)** that are tyrosine-phosphorylated from the phosphotyrosine proteomics screen.

In order to initially demonstrate that OCT1 is tyrosine phosphorylated, in a manner similar to that reported previously for OCT2 (Sprowl et al., 2016), an unbiased MS-based proteomics analysis was performed to identify all tyrosine-phosphorylated proteins, membrane-localized or intracellular, from murine tissues (Figure 1A; Supplementary Table S1). A total of 802 redundant phosphorylated peptide assignments to 438 non-redundant phosphorylated peptides for the phospho-tyrosine motif antibody were identified, applying a 5% false-positive rate to filter the results. The hits included multiple transporters (Figure 1B), including OCT1, but also several ion channels and enzymes (Figure 1C). These preliminary findings thus verified our hypothesis, suggest that tyrosine-phosphorylation may be a much more widespread regulatory mechanism of ADME proteins than held previously, and that these proteins are potentially sensitive to off-targeted de-regulation by clinically-used TKIs.

**FIGURE 2:**
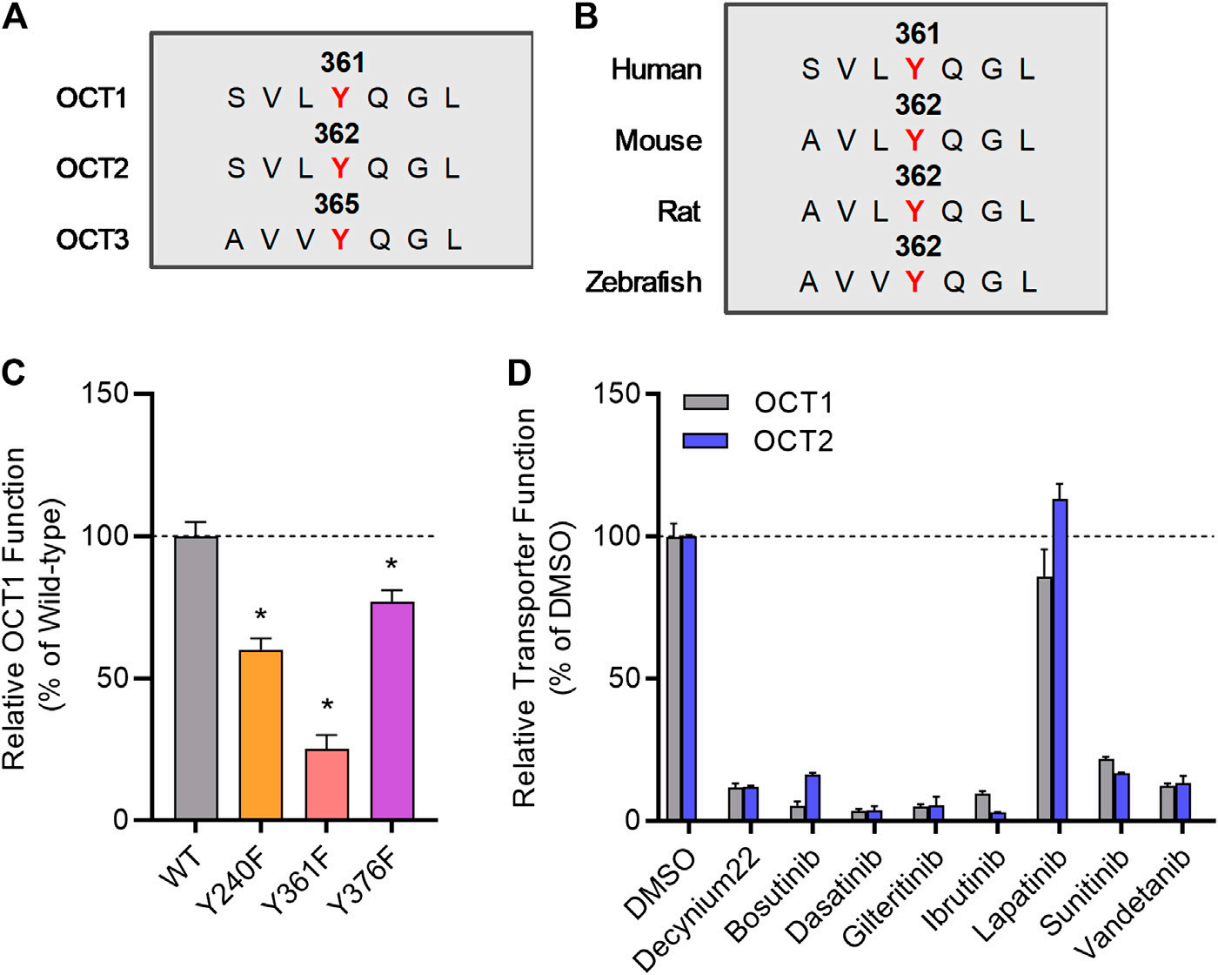
Inhibition of organic cation transporters by TKIs. **(A)** The protein sequence of hOCT1, hOCT2, and hOCT3 was aligned by a multiple sequence alignment program (MAFFT). **(B)** OCT1 protein sequence from indicated organisms was aligned by a multiple sequence alignment program (MAFFT). **(C)** HEK293 cells were transiently transfected with wild-type (WT), Y240F, Y361F, and Y376F mutant plasmids, uptake assays were performed using [^14^C] TEA (2 µM) for 15 min. Cellular accumulation of [^14^C] TEA was determined by liquid scintillation counter, and the graph represents relative uptake values compared to wild-type after normalization of protein levels. **(D)** Relative transporter function in HEK293 cells stably transfected with hOCT1 was evaluated by a substrate drug TEA in the presence of FDA-approved TKIs (10 µM) previously found to inhibit OCT2. Lapatinib was included as a negative-control TKI, and decynium22 as a non-TKI positive control inhibitor. The graph represents relative transport activity of indicated substrate drug compared to DMSO. *p < 0.05 vs. wild-type control. All values represent mean ± SEM.

We previously reported that several TKIs can modulate OCT2 function through inhibition of the protein kinase YES1, and that tyrosine-to-phenylalanine (Y-F) OCT2 mutants at three sites (241, 362, and 377) considerably diminished OCT2 function without affecting OCT2 expression in plasma membrane (Sprowl et al., 2016). In addition, OCT2 has a proline-rich (PXXPR) sequence, which is known to attach the Src Homology 3 (SH3) domain present in YES1 kinase, and mutations in this proline-rich SH3 binding domain decreased OCT2 function and tyrosine-phosphorylation. Interestingly, all these OCT2 domains, including the functionally most relevant 362 residue, are uniquely conserved in phylogenetically-linked transporters, such as OCT1, and across model organisms (Figures 2A,B). In addition, a naturally-occurring single nucleotide variant in the OCT1 gene, causing a P283L change, is known to reduce OCT1 function and alter metformin transport in humans (Mato et al., 2018), and this site is located in the proline-rich SH3 binding sequence of OCT1.

To investigate directly if the regulation of OCT2 by phosphorylation is conserved in OCT1, we performed functional assays after mutagenesis of relevant sites, and found that OCT1 mutants lacking the putative phosphorylation sites in OCT1 at residues 240, 361, and 376, corresponding to the 241, 362, and 377 sites in OCT2, had significantly reduced transport function (Figure 2C). Moreover, we found that distinct OCT2-inhibiting TKIs, including bosutinib, dasatinib, gilteritinib, ibrutinib, sunitinib, and vandetanib, but not the negative-control TKI lapatinib, also inhibit OCT1 function (Figure 2D). These results support the possible existence of a common inhibitory mechanism by which TKIs can modulate the function of OCT1 and OCT2, a conclusion, that is consistent with the notion that the OCT1- and OCT2-inhibitory properties of the studied TKIs are strongly correlated. Interestingly, compared to OCT1 and OCT2, a highly distinct TKI-mediated inhibitory profile was observed for the related transporter OCT3 (Supplementary Figure S2), with some TKIs (e.g., dasatinib, sunitinib) potently inhibiting all three transporters and some (e.g., bosutinib, gilteritinib, ibrutinib) having no influence on OCT3 function.

### TKI-Based Inhibition of OCT1 *In Vitro*

**FIGURE 3:**
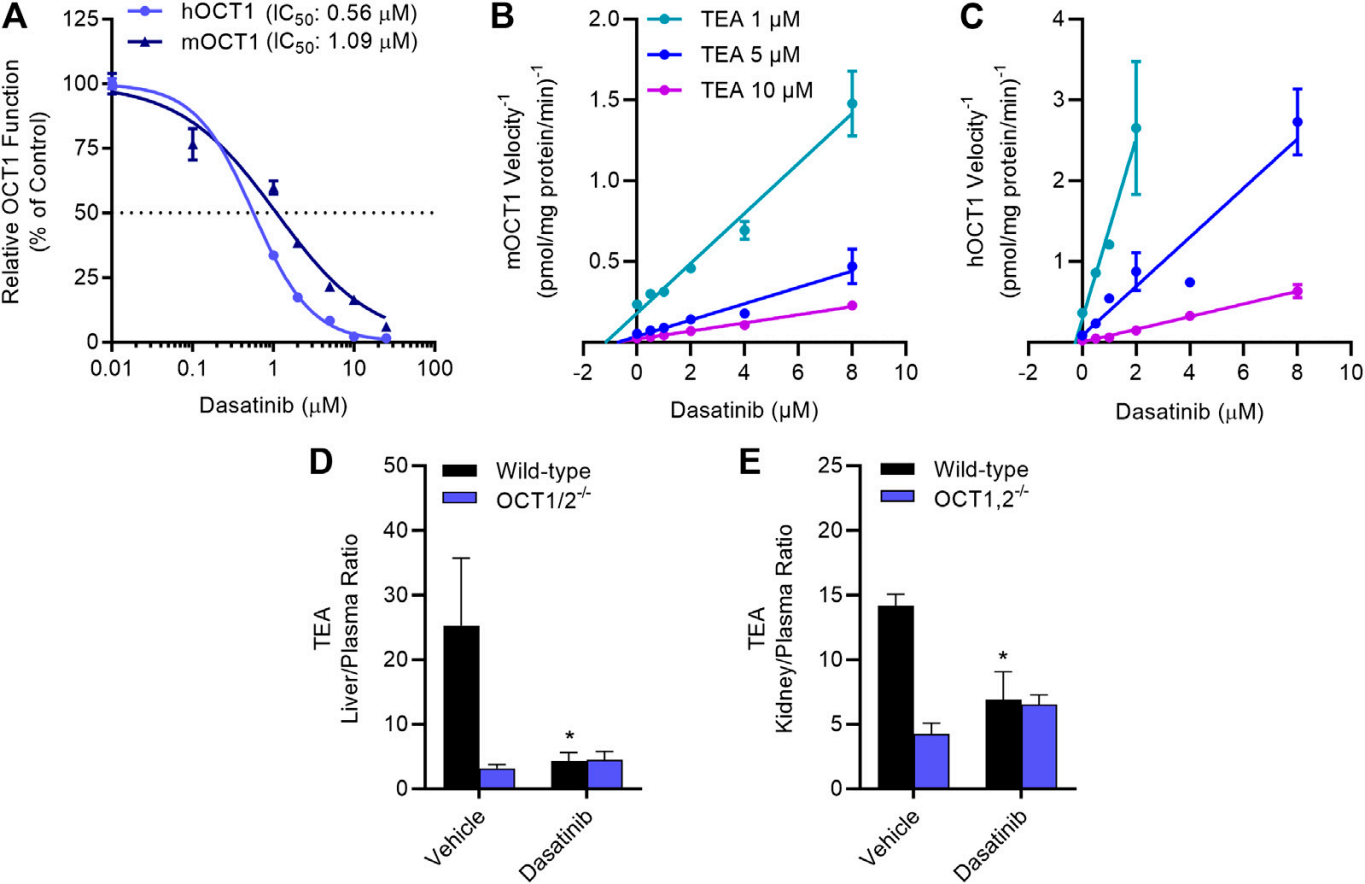
TKI-mediated inhibition of OCT1 function. **(A)** Uptake of [^14^C] TEA (2 µM) was measured in HEK293 cells overexpressing hOCT1 and mOCT1 after pre-incubation with dasatinib at various concentrations (0.1–25 µM) for 15 min, followed by the co-incubation with TEA for 15 min. Data represent the mean ± SEM and are expressed as a percentage over control. **(B,C)** Dixon plot showing varying concentrations of [^14^C] TEA (1, 5, and 10 µM) uptake assay in the presence of dasatinib (0.1–8 µM) in HEK293 cells overexpressing hOCT1 and mOCT1, data expressed as 1/velocity. In a Dixon plot, the point of intersection of the lines represent the negative inhibition constant (-Ki); this analysis revealed dasatinib-mediated inhibition constants of 0.18 µM for hOCT1 and 0.87 µM for mOCT1 (*n* = 3 per group). **(D,E)** Wild-type and OCT1/2-deficient male mice (*n* = 5) were treated with either vehicle or dasatinib (15 mg/kg) 30 min before an intravenous administration of [^14^C] TEA (0.2 mg/kg). Liver **(D)** and kidney **(E)** samples were collected at 5 min after TEA treatment, and graphed as tissue-to-plasma ratios. *p < 0.05 vs. vehicle control. All values represent mean ± SEM.

Dose-response experiments with select TKIs (Table 1) indicated that dasatinib, gilteritinib, ibrutinib, and vandetanib potently inhibited OCT1 function in a species-independent manner (Supplementary Figure S3), and regardless of the test substrate at concentrations that are clinically achievable at the recommended daily doses. Among the tested TKIs, dasatinib was found to be the most potent inhibitor against both mOCT1 (IC_50_, 1.09 μM) and hOCT1 (IC_50_, 0.56 μM) (Figure 3A), and was selected for further mechanistic studies. In line with previous observations for OCT2-inhibitory TKIs (Minematsu and Giacomini, 2011; Sprowl et al., 2016), inhibition of mOCT1 and hOCT1 by TKIs was independent of the substrate concentration, and a Dixon plot of the reciprocal velocity against the TKI concentration to derive inhibition constants indicated that the mechanism of inhibition is non-competitive (Figures 3B,C). This conclusion is consistent with our previous observation that TKs such as dasatinib are not themselves transported substrate of OCT1 (Furmanski et al., 2013).

**TABLE 1:** Features of TKIs used in the experiments.

TKI	Indication(s)	Primary target(s)	YES1 K_d_ (nM)	OCT1 IC_50_ (µM)	OCT1 inhibition	OCT2 inhibition
Dasatinib	CML, GIST	BCR/ABL, SRC	0.3	0.56–1.09	Yes	Yes
Gilteritinib	AML	FLT3, AXL	445	0.01–0.02	Yes	Yes
Ibrutinib	CLL, MCL	BTK	27	0.89–1.18	Yes	Yes
Lapatinib	Breast cancer	HER2, EGFR	>10,000	—	No	No
Vandetanib	Thyroid cancer	EGFR, VEGFR	120	1.35–9.05	Yes	Yes

CLL, chronic lymphocytic leukemia; CML, chronic myeloid leukemia; GIST, gastrointestinal stromal tumor; MCL, mantle cell lymphoma; AML, acute myeloid leukemia.

### TKI-Mediated Modulation of OCT1 *In Vivo*

The notion that the OCT1-inhibitory properties of dasatinib are species-independent is consistent with and recapitulates several prior observations (Shu et al., 2007; Sprowl et al., 2016; Floerl et al., 2020; Meyer et al., 2020), and suggests that mice can serve as a suitably predictive model for humans. To directly assess the influence of dasatinib on the function of OCT1 *in vivo*, the pharmacokinetic profile of TEA was examined in wild-type mice and OCT1/2-deficient mice receiving a single oral dose of dasatinib, given 30 min before the administration of TEA. We found that the hepatic uptake of TEA, as determined from the liver-to-plasma concentration ratio, was dramatically reduced in the OCT1/2-deficient mice, and that the genotype could be phenocopied by a single dose of dasatinib (Figure 3D). Similar observations were made in the murine kidney (Figure 3E), an organ that expresses both OCT1 and OCT2 (Holle et al., 2011).

**FIGURE 4:**
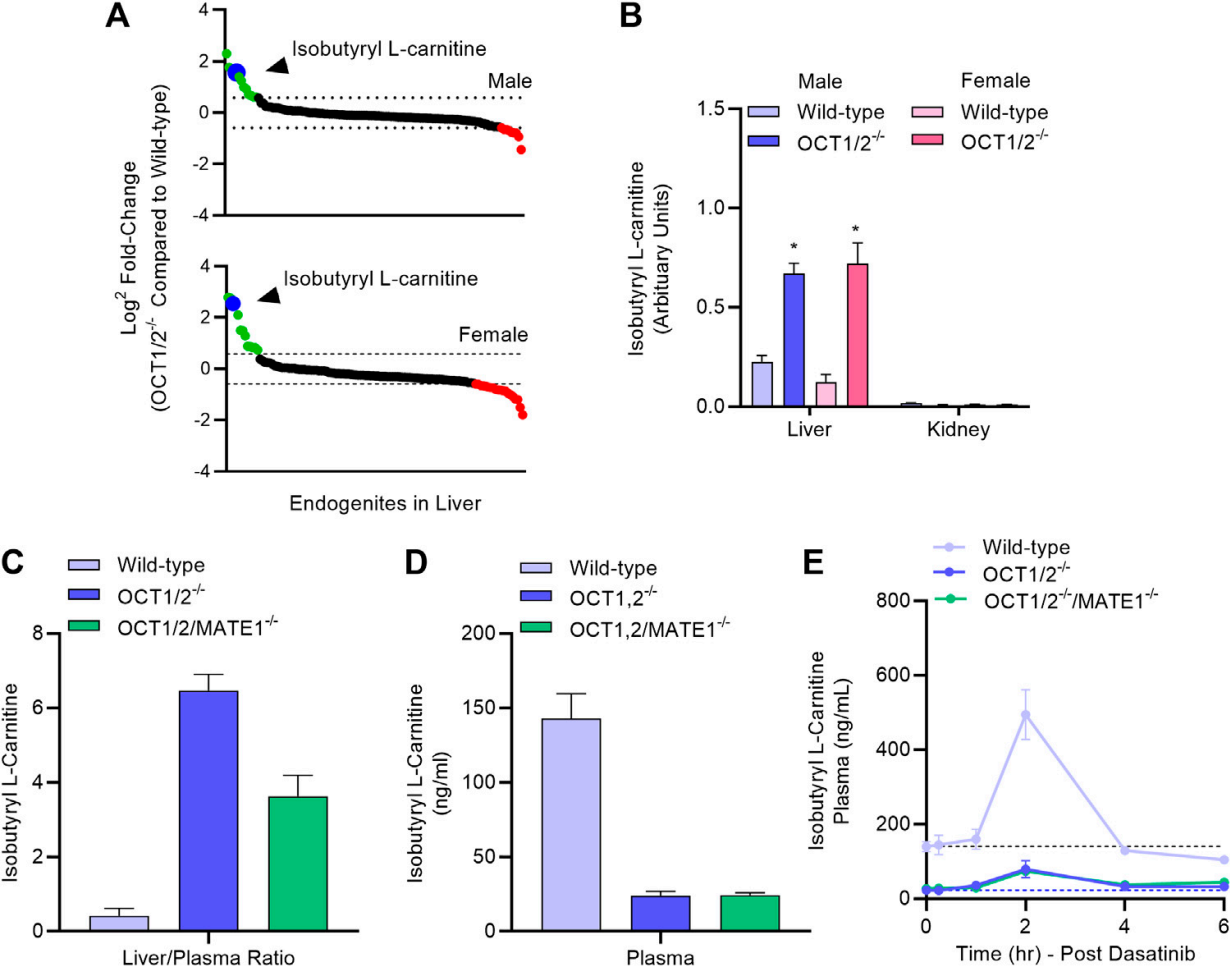
Targeted metabolomics and endogenous OCT1 biomarker identification. **(A)** Differentially quantitated endogenous metabolites (“endogenites”) in the liver of male and female wild-type mice and OCT1/2-deficient mice. Endogenites highlighted in green and red were significantly increased and decreased, respectively in livers of OCT1/2-deficient mice. The blue symbol represents isobutyryl L-carnitine (IBC). **(B)** Liver and kidney concentrations of IBC in wild-type and OCT1/2-deficient mice (*n* = 5). **(C,D)** Liver-to-plasma ratio and plasma level of IBC at baseline in wild-type mice, OCT1/2-deficient mice, and OCT1/2/MATE1-deficient mice (*n* = 5). **(E)** Plasma concentration-time profile of IBC in wild-type mice, OCT1/2-deficient mice, and OCT1/2/MATE1-deficient mice (*n* = 5) after a single oral dose of dasatinib (15 mg/kg). *p < 0.05 vs. wild-type. All values represent mean ± SEM.

In order to provide further evidence that the ability of dasatinib to modulate TEA disposition is causally related to modulation of hepatic OCT1, we next performed an LC-MS/MS-based targeted metabolomics study in samples from wild-type mice and OCT1/2-deficient mice that was designed to identify a liver-specific endogenous biomarker of OCT1. This study revealed that among 121 metabolites examined, the hepatic concentration of several compounds, including isobutyryl-l-carnitine (IBC), was substantially elevated in OCT1/2-deficient mice compared to wild-type mice (Figure 4A; Supplementary Table S2), in both male and female animals. We also observed that reduced hepatic levels of IBC in wild-type mice were accompanied by significantly elevated levels in plasma (Figure 4B), that IBC levels in the kidney were negligible (Figure 4B) regardless of mouse genotype, and that additional deficiency of MATE1 (Figure 4C), which forms a functional unit with OCT1 in the liver and with OCT2 in the kidney, did not influence the results. These findings suggest that IBC, a natural four-carbon acylcarnitine involved in fatty acid oxidation and organic acid metabolism, serves as a *bona fide* biomarker for hepatic OCT1 function, a conclusion, that is in line with a recent clinical report (Luo et al., 2020). We next evaluated the impact of dasatinib on concentrations of IBC and found that administration of the TKI resulted in a transient, statistically significant increase in the plasma levels of IBC in wild-type mice, but not in OCT1/2-deficient mice or OCT1/2/MATE1-deficient mice (Figure 4D). Taken together, these data indicate that dasatinib, given at a dose that affects the liver uptake of TEA, causes significant inhibition of hepatic OCT1 function.

### Kinase-Mediated Regulation of OCT1 Function

**FIGURE 5:**
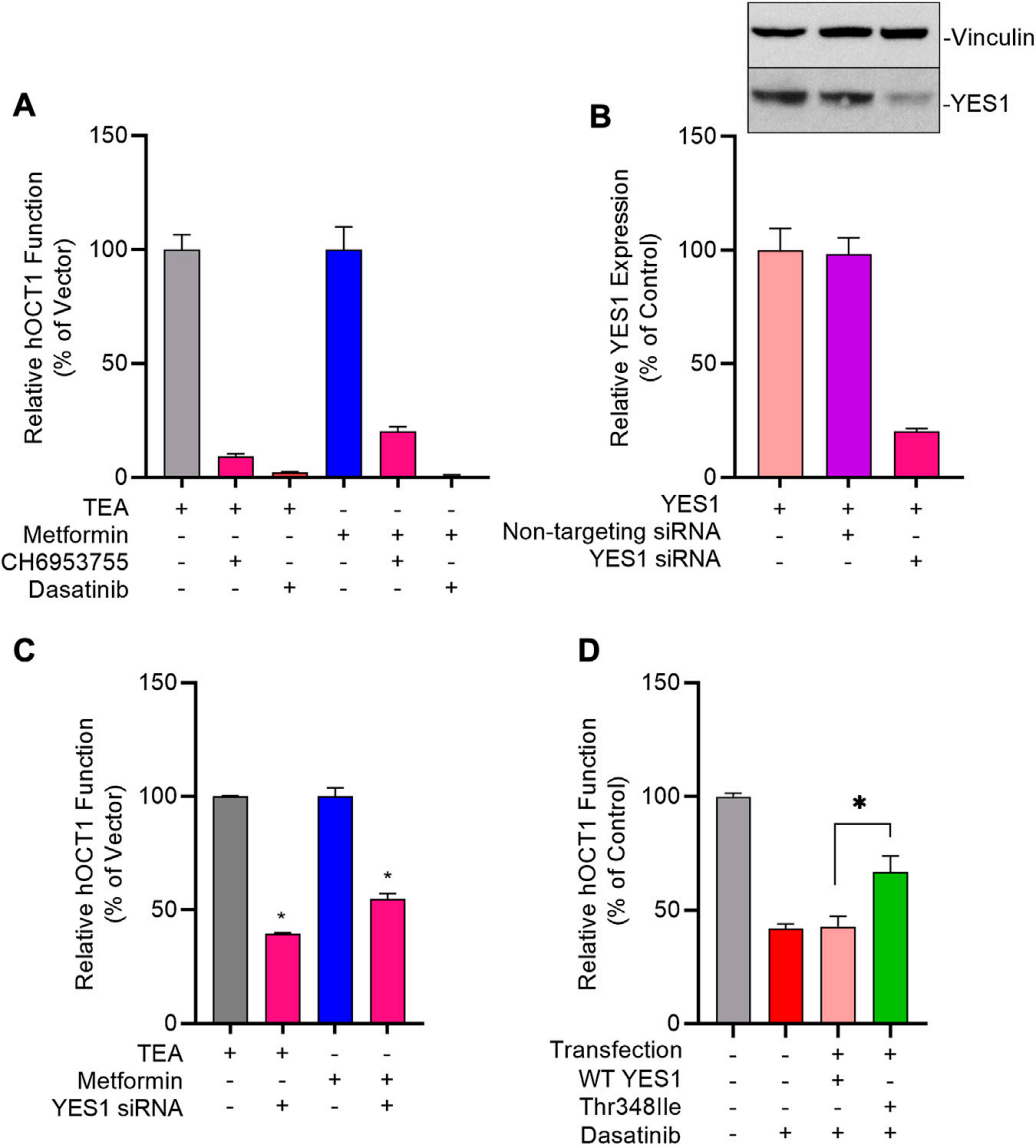
Genetic and pharmacological inhibition of YES1 kinase impairs OCT1 activity. **(A)** HEK293 cells stably transfected with vector control (VC) and hOCT1 were pre-incubated with CH6953755 or dasatinib (10 µM) for 15 min followed by the co-incubation with [^14^C] TEA (2 µM) or [^14^C] metformin (5 µM). Data represents relative uptake values compared to VC control after normalization of protein levels. **(B)** Expression of YES1 protein (top) and gene (bottom) in hOCT1-expressing HEK293 cells 48 h after transfection with non-targeting siRNA or YES1 siRNA. **(C)** Influence of YES1 silencing by siRNA on hOCT1 function was measured in HEK293 cells using uptake assays with [^14^C] TEA or [^14^C] metformin. **(D)** Influence of YES1 mutants on dasatinib-mediated inhibition of hOCT1 function in HEK293 cells following transient transfection of constructs carrying either wild-type YES1 or the YES1 Thr348Ile gatekeeper mutant. After 48 h, cells were pre-treated with dasatinib (1 μM) for 15 min, followed by uptake assay using [^14^C] TEA. *p < 0.05 vs. control. All values represent mean ± SEM.

The existence of tyrosine motifs that are conserved between OCT1 and OCT2, and the similarity in sensitivity to inhibition by TKIs between these two transporters raises the possibility that the tyrosine phosphorylation and activity of OCT1 are regulated by YES1, as described for OCT2 (Sprowl et al., 2016). In support of this hypothesis, we found that pre-treatment of OCT1-expressing cells with the selective YES1 inhibitor, CH6953755, causes substantial inhibition of hOCT1-mediated transport of TEA (IC_50_, 2.76 µM) and metformin (IC_50_, 2.31 µM) (Figure 5A; Supplementary Figures 4A,B). This degree of inhibition by CH6953755 was also observed in models overexpressing mOCT1 (Supplementary Figure S4C) or hOCT2 (Supplementary Figure S4D). The connection of TKI-mediated OCT1 inhibition with the function of YES1 was further substantiated by the observed relationship between potency of target engagement by the studied TKIs, as determined by the affinity constant (K_d_) (Klaeger et al., 2017; KINOMEscan data—HMS LINCS Project, 2020), and their ability to modulate OCT1-mediated transport (Supplementary Figure S4E).

To demonstrate causality of this relationship, we found that even partial downregulation of YES1 expression by siRNA in HEK293 cells (Figure 5B) was already associated with a statistically significant loss of OCT1 transport function (Figure 5C). To unambiguously identify YES1 as the TKI-sensitive protein kinase that phosphorylates OCT1, we next carried out a screen utilizing a chemical genetics approach in which HEK293 cells expressing hOCT1 are transfected with either the wild-type or TKI-resistant (T348I gatekeeper) mutant of YES (Du et al., 2009; Li et al., 2010), followed by dasatinib treatment and OCT1 uptake assays (Supplementary Figure S5). These studies revealed that the TKI-resistant YES1 mutant was able to rescue OCT1 inhibition by dasatinib, whereas cells carrying the YES1 wild-type construct retained sensitivity to dasatinib-mediated OCT1 inhibition (Figure 5D).

## Discussion

In the present study, we identified OCT1 as a tyrosine-phosphorylated transporter from a phospho-proteomics screen, and demonstrated through functional validation studies using genetic and pharmacological approaches that OCT1 is highly sensitive to small molecules in the class of TKIs that target the protein kinase YES1, such as dasatinib. In addition, we found that dasatinib can inhibit hepatic OCT1 function in mice as evidenced from its ability to modulate levels of the prototypical substrates TEA and metformin as well as the OCT1 endogenous biomarker, isobutyryl L-carnitine. These findings provide novel insight into the posttranslational regulation of OCT1 and suggest that caution is warranted with polypharmacy regimes involving the use of OCT1 substrates in combination with TKIs that target YES1 (Minematsu and Giacomini, 2011; Lautem et al., 2013; Jensen et al., 2020). This is particularly relevant in view of the fact that more than one-third of approved prescription drugs are positively charged at neutral pH, and that the membrane transport of many of these agents relies on facilitated carriers such as OCT1.

Previous studies have indicated that OCT1 expression is regulated at different levels, including transcriptionally, by intracellular trafficking, and through alteration of functional properties. Among these mechanisms, transcriptional regulation by hepatic nuclear factors (HNF1 and HNF4α) has been well documented. This work has suggested that HNF1 ties to an evolutionary conserved region within intron 1 (O’Brien et al., 2013), whereas HNF4α is involved in bile acid-dependent regulation of OCT1 in the liver *via* activation by the bile acid-inducible transcriptional repressor (Saborowski et al., 2006). In addition, OCT1 expression can be regulated by hepatic growth factor (Le Vee et al., 2009), and activity of the OCT1 promoter is affected by methylation (Shu et al., 2007; Schaeffeler et al., 2011; Mato et al., 2018).

In contrast to this knowledge on transcriptional mechanisms, details of short-term posttranslational regulation of OCT1 activity have remained incompletely understood. It was previously reported that substrate transport of OCT1 is reduced by activation of protein kinase A and by inhibition of calmodulin, CaM-dependent kinase II, or p56lck tyrosine kinase (Ciarimboli et al., 2005). Our current findings add to this prior knowledge and demonstrate that many ADME proteins, including multi-specific drug-transporters such as OCT1, are directly regulated through tyrosine-phosphorylation by a mechanism that involves the kinase YES1 in a manner, that is analogous to that previously reported for OCT2 (Sprowl et al., 2016). Our study also indicates that disruption of this phosphorylation process by YES1 by several clinically-used TKIs can result in dramatically impaired OCT1 function. Furthermore, our study suggests that phospho-proteomic analysis should be considered during the drug development process to predict potential drug-drug interactions and to avoid unwanted consequences when potent inhibitors of YES1 kinase are administered together with agents that undergo OCT1-dependent hepatic transport (Tzvetkov et al., 2013; Matthaei et al., 2016).

During the course of our investigation, we identified several FDA-approved TKIs as previously unrecognized, potent inhibitors of OCT1, including dasatinib, ibrutinib, sunitinib, and vandetanib. In addition, we confirmed the OCT1-inhibitory potential of several other TKIs, such as bosutinib and gilteritinib, which are listed as OCT1 inhibitors in their prescribing information. It should be pointed out that the mechanism by which these agents impede OCT1 transport function is not distinctly illustrated in the prescribing information of most TKIs (e.g., reversible *vs*. irreversible; non-competitive vs. competitive). The presence or absence of either pre- and co-incubation of TKIs with probe substrates could influence on the inhibitory potential toward transporters, and lead to false-negative results. For example, addition of dasatinib in pre-incubation conditions potently inhibits OCT2 function in experimental studies (Sprowl et al., 2016), whereas co-incubation designs, based on an *a priori* presumed competitive mechanism of inhibition, dasatinib was identified as only a weak inhibitor of OCT2, that is unlikely to have *in vivo* relevance (Minematsu and Giacomini, 2011). Because of the discrepancies in published reports and prescribing information, we have previously argued that a reliable and reproducible approach needs to be implemented to explicitly determine TKI-transporter interactions with a statistically meaningful and unbiased manner is essential in order to evade contradictory results, and should ultimately be applied for the design of subsequent *in vivo* validation studies (Huang et al., 2020). In addition, variations among different laboratory settings, including selection of the test substrate(s) (Sandoval et al., 2018), demand that choosing appropriate model substrates should become an essential component in conducting *in vitro* cationic-type transport assays to generate useful and translationally-relevant predictions.

The lack of regulatory guidelines on the experimental design and clarification of *in vitro* studies to determine an inhibitory potential of drugs with transporters has likely influenced many of the reported inconsistencies. Since TKI agents are most frequently prescribed as a chronic treatment (e.g., once or twice daily) along with numerous other medications, it is anticipated that researchers will be vigilant regarding the potential transporter-mediated drug-drug interactions of TKIs as a perpetrators in order to achieve new mechanistic insights and to enhance the safety of currently used polypharmacy regimens. One approach explored in our current study to demonstrate direct *in vivo* modulation of hepatic OCT1 function following the administration of dasatinib is through the identification of novel biomarkers that could ultimately be utilized to guide the selection of optimal doses and schedules of potential perpetrators to be used in conjunction with OCT1 substrates. This was accomplished by probing for novel endogenous metabolites of OCT1 that reflect hepatic transport function and that can be detected in the circulation, by conducting targeted MS-based metabolomic analyses. This analysis was performed using plasma and liver specimens from wild-type mice and OCT1/2-deficient mice, and ultimately resulted in the identification of various structurally named molecules of possible significance, including isobutyryl-l-carnitine (IBC). Interestingly, while we were completing the current study, Luo et al. reported that IBC is also a potentially useful endogenous biomarker to predict OCT1-mediated drug-drug interactions in humans (Luo et al., 2020). These collective findings are largely congruent with prior studies by Kim et al. on the transport of carnitines in liver-specific OCT1-knockout mice (Kim et al., 2017). This work suggests that levels of certain short-chain acylcarnitines are increased in livers of OCT1-deficient mice but unchanged in plasma, and also that OCT1 serves to efflux carnitines out of cultured hepatocytes but not to take them up. This is consistent with the prior observation that acylcarnitines are not taken up by cells engineered to overexpress OCT1 (Lancaster et al., 2010). In our metabolomics data, we did not observe apparent changes in the levels of IBC in the kidney, where OCT2 is most highly expressed, and we found that additional deficiency of MATE1 had no influence on the results. Although the baseline differences of IBC in plasma between wild-type mice and the various OCT1-deficient strains suggests that levels are predominantly influenced by OCT1-mediated hepatic efflux, we found that dasatinib administration to wild-type mice was actually associated with an increase in the levels of IBC in plasma. Although this observation seems counterintuitive, it should be noted that OCT1 can serve as a bi-directional hepatic transporter to either mediate the electrogenic cellular influx or alternatively to mediate efflux of organic cations under *trans*-zero conditions, depending on the substrate concentration gradient. Regardless of the mechanistic basis, the recorded discrepancy with the recently published human study (Luo et al., 2020) suggests that further investigation into the use of IBC as an OCT1 biomarker in the context of transport inhibitors is warranted.

In conclusion, we identified a novel regulatory mechanism for OCT1 function that involves tyrosine phosphorylation by the kinase YES1, and, that is highly sensitive to inhibition by multiple TKIs, including dasatinib. OCT1 is highly expressed in hepatocytes and plays a crucial role in the elimination and pharmacological activity of many prescription drugs, and this makes OCT1 uniquely vulnerable to phosphorylation-mediated interactions with TKIs.

## Data Availability

The datasets presented in this study can be found in online repositories. The names of the repository/repositories and accession number(s) can be found below: https://www.ebi.ac.uk/metabolights/MTBLS2433; https://datadryad.org/stash/share/iV95lgOy_VGJLpUReX4ukg77I7vcJueCEwJ0S5p4a0Y.
