# Supplementary figures and images for: Influence of YES1 Kinase and Tyrosine Phosphorylation on the Activity of OCT1
Source: Front Pharmacol. 2021 Mar 8;12:644342. doi: 10.3389/fphar.2021.644342 (PMC8006202; doi:10.3389/fphar.2021.644342)

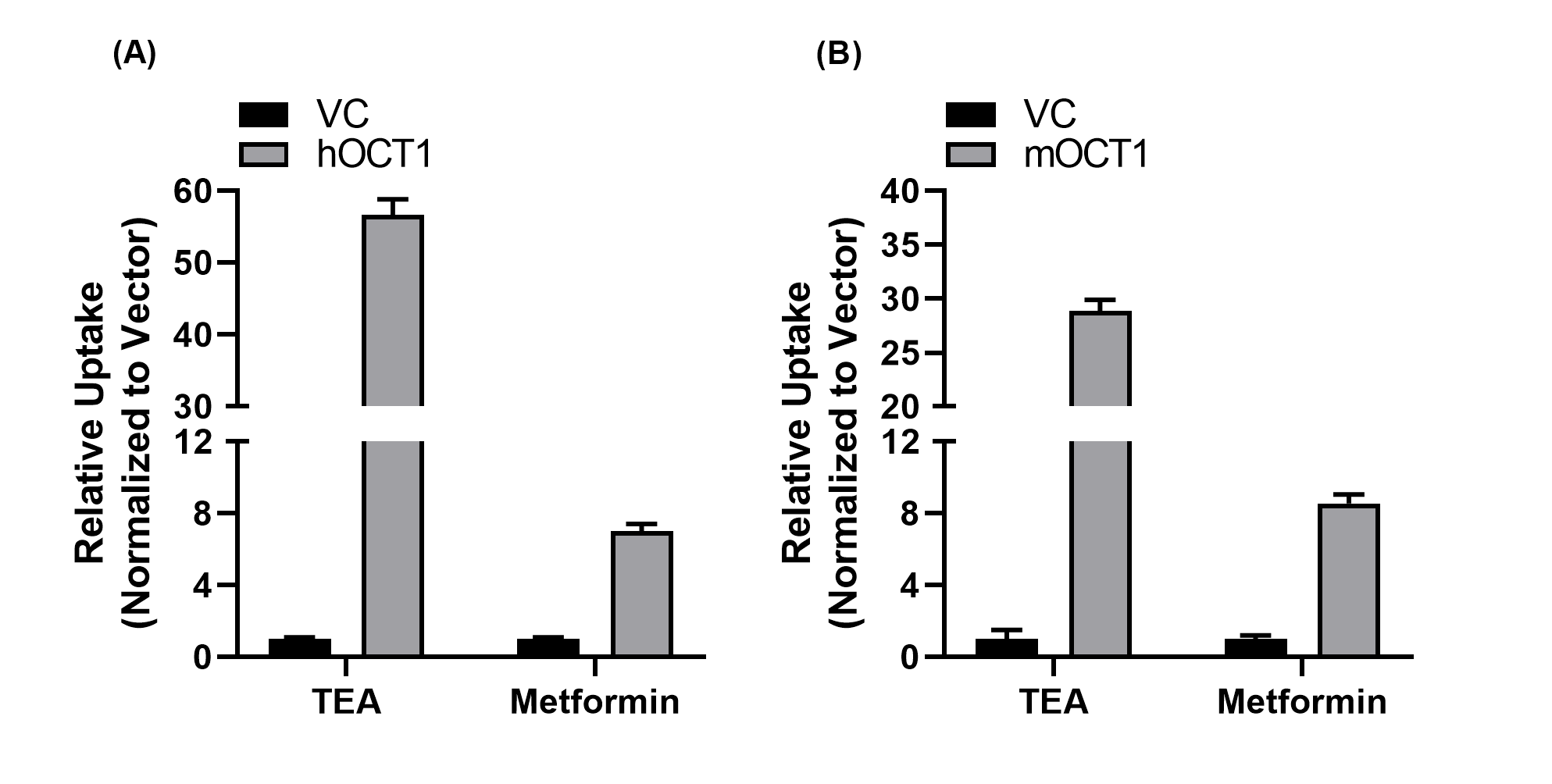

Supplement: Supplementary file 3 [file image1.tif]

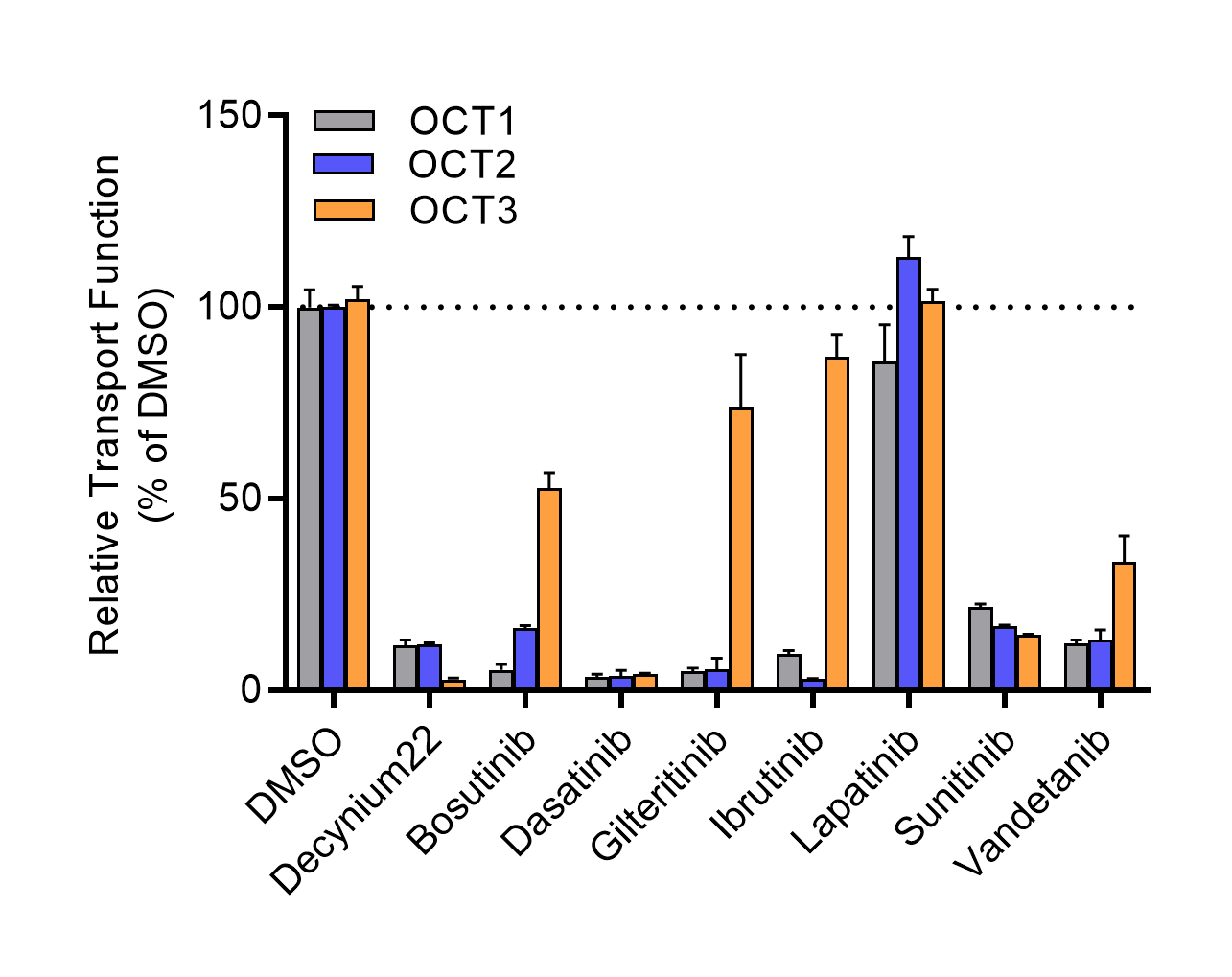

Supplement: Supplementary file 4 [file image2.tif]

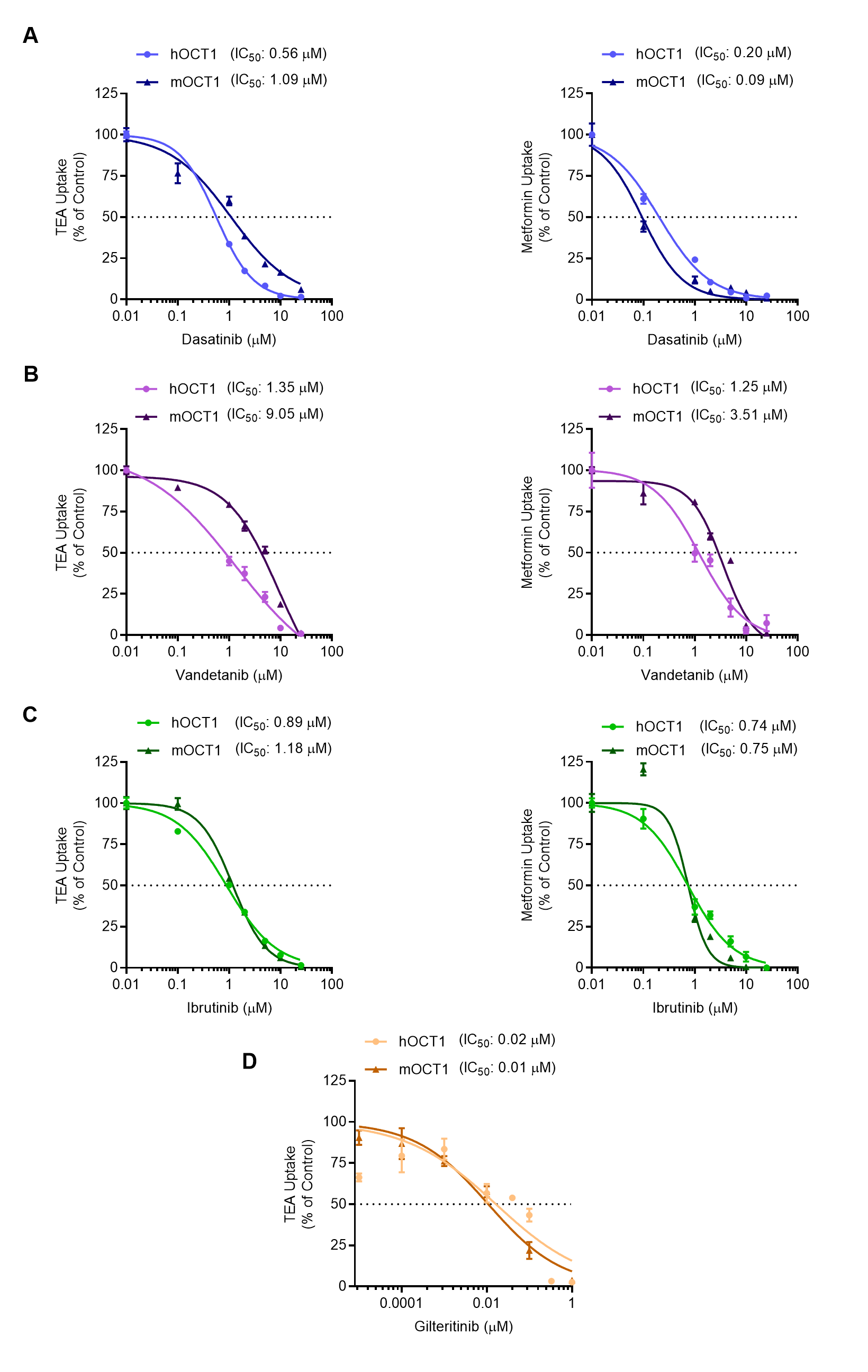

Supplement: Supplementary file 5 [file image3.tif]

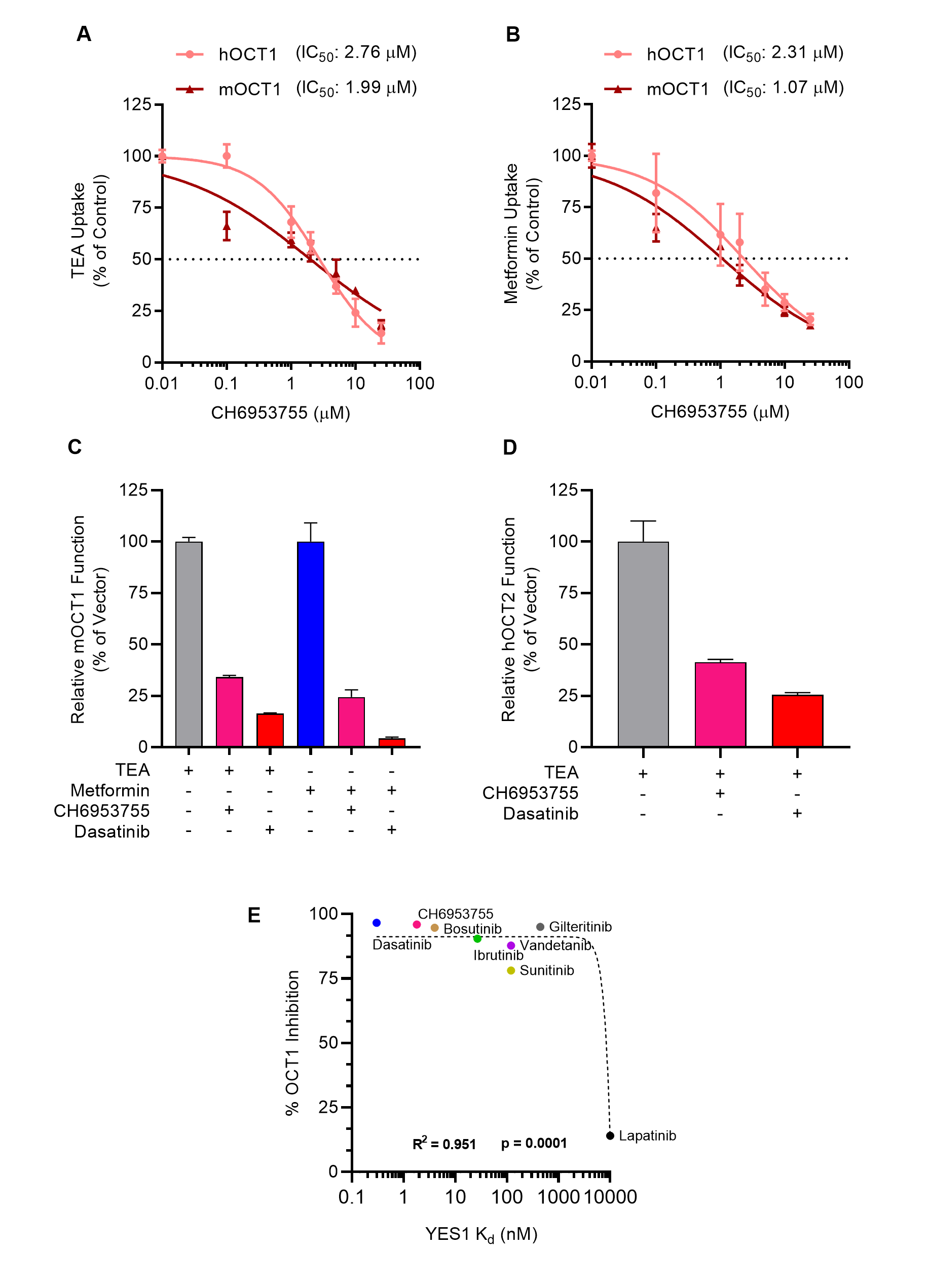

Supplement: Supplementary file 6 [file image4.tif]

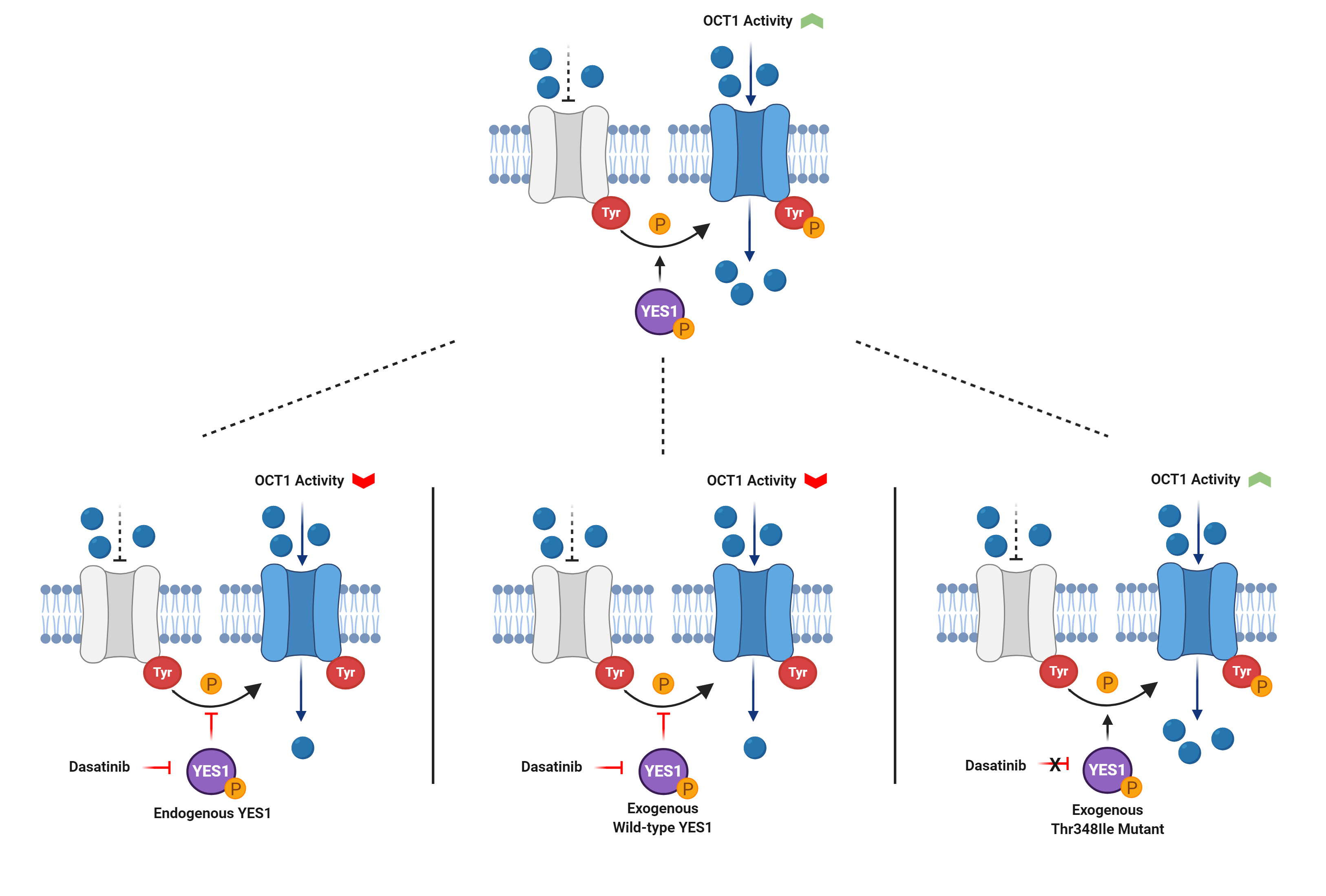

Supplement: Supplementary file 7 [file image5.jpeg]
